# Disorders Mimicking Wilson’s Disease: Clinical, Biochemical, and Molecular Perspectives for Accurate Differential Diagnosis

**DOI:** 10.3390/diagnostics16091342

**Published:** 2026-04-29

**Authors:** Agnieszka Antos, Grażyna Gromadzka, Jan Paweł Bembenek, Tomasz Litwin

**Affiliations:** 1 Second Department of Neurology, Institute of Psychiatry and Neurology, 02-957 Warsaw, Poland; 2 Department of Biomedical Sciences, Faculty of Medicine, Collegium Medicum, Cardinal Stefan Wyszynski University, 01-938 Warsaw, Poland; gragrom@gmx.com; 3 Department of Clinical Neurophysiology, Institute of Psychiatry and Neurology, 02-957 Warsaw, Poland; jbembenek@ipin.edu.pl; 4 Department of Neurology, Wolski Hospital, 01-211 Warsaw, Poland; tomlit@medprakt.pl

**Keywords:** Wilson’s disease, aceruloplasminemia, copper dysregulation, copper metabolism, hepatic disorders, genetic testing

## Abstract

Wilson’s disease (WD) is an autosomal recessive disorder of copper metabolism caused by *ATP7B* mutations, characterized by hepatic copper accumulation and multisystem involvement. Several rare inherited and acquired conditions can closely mimic WD, posing diagnostic challenges and the risk of inappropriate therapy. By examining neuroimaging patterns and distinguishing between diagnostic criteria, this narrative review provides a comprehensive synthesis of WD-mimicking disorders, emphasizing their molecular mechanisms, clinical phenotypes, and biochemical features. WD-mimicking disorders encompass *ATP7A*-related neurodegenerations (Menkes disease, occipital horn syndrome, X-linked distal hereditary motor neuropathy), MEDNIK syndrome, Huppke–Brendel syndrome, aceruloplasminemia, congenital disorders of glycosylation, primary familial intrahepatic cholestasis type 3, and acquired copper deficiency syndromes. Mechanisms include systemic copper deficiency, impaired intracellular trafficking, defective ceruloplasmin biosynthesis, secondary hepatic copper accumulation, and abnormal glycosylation. Clinical features range from neurodevelopmental delay, movement disorders, and hepatic dysfunction to dermatologic, hematologic, and connective-tissue abnormalities. Biochemical profiles may overlap with WD, particularly low serum ceruloplasmin and total copper, altered urinary copper excretion, and elevated hepatic copper in some disorders. Neuroimaging and genetic testing provide critical discriminative value. Management is largely supportive, with disease-specific therapies available in selected conditions, such as subcutaneous copper in Menkes disease or monosaccharide supplementation in certain congenital disorders of glycosylation subtypes. Accurate differentiation between WD and WD-mimicking disorders requires careful integration of clinical, biochemical, imaging, and molecular data. Recognition of distinctive features and understanding underlying pathophysiology are essential to avoid misdiagnosis and inappropriate anti-copper therapy, optimize management, and improve patient outcomes.

## 1. Introduction

### 1.1. Wilson’s Disease

**Figure 1:**
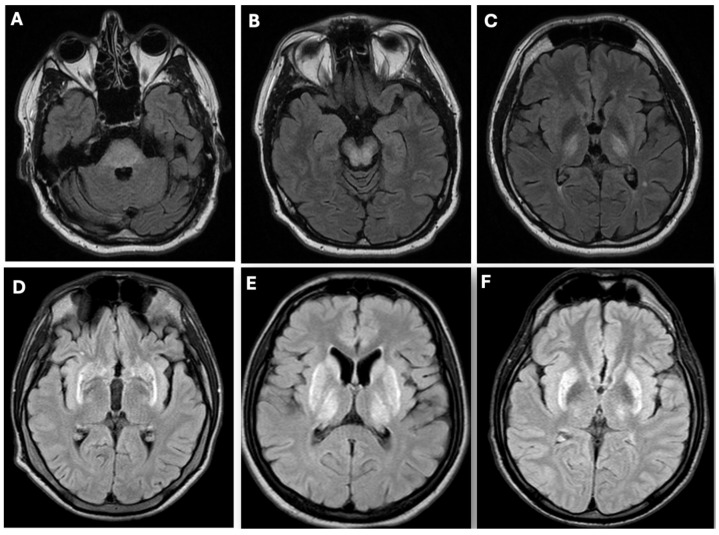
Brain MRI pathology in Wilson’s disease—symmetrical hyperintense signal in FLAIR sequences in: (**A**) pons, (**B**) midbrain, (**C**) putamen and thalamus, (**D**) putamen, (**E**) putamen, thalamus and head of nucleus caudate, (**F**) putamen and thalamus (authors’ material from the Department of Neurology).

Wilson’s disease (WD; OMIM #277900) is an autosomal recessive disorder of copper metabolism caused by pathogenic variants in the *ATP7B* gene (OMIM *606882) [1,2,3]. The encoded ATPase 7B is a transmembrane copper-transporting ATPase essential for incorporating copper into apoceruloplasmin and excreting excess copper into bile. Abnormal or lost *ATP7B* function disrupts these processes, leading to toxic hepatic copper accumulation, oxidative stress, and progressive multiorgan injury [1,2,3]. As hepatic copper overload exceeds the hepatocyte’s buffering capacity, toxic non-ceruloplasmin (Cp)-bound copper (NCC, or “free” copper) is released into the circulation and accumulates in extrahepatic tissues, particularly the central nervous system and cornea [4]. This mechanism underlies the characteristic multisystem involvement of WD [3]. Neurological symptoms—including tremor, dystonia, chorea, parkinsonism, athetosis, dysarthria, sialorrhea, dysphagia, and gait or postural disturbances—are often the most disabling [1,2,3,4,5]. Psychiatric manifestations are also common and may encompass mood disorders, behavioral dysregulation, personality changes, and cognitive impairment [1,2,3,6]. Brain magnetic resonance imaging (MRI) may demonstrate symmetrical basal ganglia and mesencephalon involvement (Figure 1) [1,2,3,7].

Ophthalmologic findings such as Kayser–Fleischer rings and sunflower cataracts are highly suggestive of copper overload. Additional organ involvement, including renal tubular dysfunction, endocrine abnormalities, and Coombs-negative hemolytic anemia, reflects the systemic toxicity of free copper [1,2,3].

WD is one of the few inherited metabolic diseases for which effective disease-modifying therapy exists [1,8,9,10,11,12,13,14,15]. Copper-lowering treatment aims to achieve a negative copper balance through chelators (D-penicillamine, trientine) or zinc salts that inhibit intestinal copper absorption [1,2,3]. Early initiation and sustained adherence are crucial, with approximately 85% of patients demonstrating hepatic or neurological improvement or stabilization of disease progression [1,2,3].

Diagnosis relies on an integrated assessment of biochemical, clinical, and genetic findings [11,15]. Key laboratory abnormalities include low serum Cp and total serum copper, elevated 24 h urinary copper excretion, and increased hepatic copper concentration confirmed by liver biopsy. Molecular testing for *ATP7B* variants increases diagnostic certainty. These parameters are combined in the Leipzig scoring system (Table 1), which standardizes diagnostic evaluation [11].

However, several rare inherited or acquired disorders of copper metabolism—or conditions that secondarily disrupt copper handling—may closely mimic WD, both clinically and biochemically [16]. These disorders can present with hepatic failure, neurological syndromes, psychiatric symptoms, or combinations thereof, creating substantial potential for misdiagnosis and inappropriate initiation of anti-copper therapy. Given the significant therapeutic and prognostic consequences, accurate differentiation between WD and WD-mimicking disorders is essential [1,12,17].

### 1.2. Aim of the Review

The aim of this review was to provide a comprehensive and clinically oriented synthesis of disorders that mimic WD, with a focus on their molecular mechanisms, clinical phenotypes, biochemical signatures, and distinguishing diagnostic features. By comparing WD with inherited and acquired conditions characterized by copper dysregulation or overlapping hepatic, neurological, and psychiatric manifestations, this review seeks to support accurate differential diagnosis and prevent misclassification leading to inappropriate therapy.

### 1.3. Review Methodology

This narrative review was conducted to synthesize current knowledge on disorders that mimic WD, with an emphasis on their genetic basis, pathophysiology of copper handling, clinical presentations, biochemical and neuroimaging characteristics, and diagnostic pitfalls. A comprehensive literature search was performed in PubMed, Scopus, and Web of Science databases from inception to January 2025.

The following keywords and their combinations were used: “Wilson’s disease”, “*ATP7B*”, “copper metabolism”, “copper transport disorders”, “Cp”, “aceruloplasminemia”, “*CP* gene”, “Menkes disease”, “*ATP7A*”, “occipital horn syndrome”, “distal hereditary motor, x-linked, neuropathy”, “HMNX”, “HPBDS”, “*SLC33A1*”, “copper toxicity”, “differential diagnosis”, “hepatic failure”, “neuropsychiatric symptoms”, “movement disorders”, “Kayser–Fleischer rings”, “free copper”, “copper deficiency”, “iron metabolism disorders”, “NBIA”, “misdiagnosis”. Both original research articles and review papers published in English were included. Priority was given to peer-reviewed studies providing mechanistic, genetic, or clinically relevant diagnostic insights. Preprints, non-peer-reviewed sources, and conference abstracts were excluded. Reference lists of selected articles were manually screened to identify additional relevant publications.

Given the rarity and heterogeneity of WD-mimicking disorders, particular attention was devoted to case series, genotype–phenotype studies, mechanistic investigations of copper and iron metabolism, and recent reviews published after 2020. Extracted data were critically evaluated and synthesized into thematic sections highlighting shared clinical features, biochemical clues for differentiation, and remaining gaps in diagnostic practice.

The copper metabolism disorders described below are presented in accordance with the physiological pathway of human copper metabolism—from enterocytes, through hepatocytes, and other cellular compartments—as well as their etiology, rather than based on their clinical similarity to WD.

## 2. Copper Metabolism Disorders Mimicking Wilson’s Disease

### 2.1. ATP7A-Related Copper Neurodegenerations

The *ATP7A* gene (OMIM *300011), located on the X chromosome (Xq21.1), encodes *ATP7A*, a transmembrane copper-transporting ATPase situated in the trans-Golgi network. *ATP7A* is responsible for trafficking copper from the Golgi apparatus to the plasma membrane, enabling its export from cells and proper distribution to multiple cuproenzymes. Pathogenic variants in *ATP7A* disrupt this process, leading to systemic copper deficiency: copper is poorly absorbed from enterocytes, resulting in low serum copper levels, and fails to cross the blood–brain barrier, causing markedly reduced copper content in the brain [18,19,20].

Consequently, numerous copper-dependent enzymes become dysfunctional. These include dopamine-β-hydroxylase (impaired catecholamine synthesis), cytochrome c oxidase (mitochondrial dysfunction, demyelination, and brain atrophy), lysyl oxidase (defective collagen and elastin crosslinking), tyrosinase (hypopigmentation), and superoxide dismutase (increased oxidative stress), among others. The resulting clinical phenotype reflects the degree of *ATP7A* dysfunction and the severity of copper deprivation in individual tissues [18,19,20].

Currently, three distinct *ATP7A*-related neurodegenerative syndromes are recognized: classic Menkes disease, occipital horn syndrome and distal hereditary neuropathy. These disorders span a spectrum from severe infantile neurodegeneration to milder connective-tissue-predominant phenotypes and slowly progressive adult-onset motor axonopathy [18,19,20].

#### 2.1.1. Menkes Disease

Menkes disease (MD; OMIM #309400), also known as kinky hair disease, represents the most severe phenotype among disorders caused by pathogenic variants in the *ATP7A* gene [21,22,23]. These variants typically result in near-complete loss of *ATP7A* function, leading to profound systemic copper deficiency and widespread impairment of copper-dependent enzymatic processes. MD is a rare X-linked recessive condition, occurring in approximately 1 in 100,000 live births [20,24,25,26]. Loss of *ATP7A* function severely disrupts intestinal copper absorption, resulting in markedly reduced serum copper and Cp concentrations. Because copper fails to reach the central nervous system, brain copper levels become critically low, impairing multiple essential cuproenzymes, including dopamine-β-hydroxylase, cytochrome-c oxidase, lysyl oxidase, tyrosinase, and superoxide dismutase [19,20,20,21,22,23]. These enzymatic defects produce autonomic abnormalities, mitochondrial dysfunction, hypomyelination, connective-tissue fragility, hypopigmentation, and heightened susceptibility to oxidative injury. The consequence is a rapidly progressive multisystem disorder that overwhelmingly affects the developing nervous system [25].

MD usually presents in early infancy. Neurological involvement dominates the clinical course, with profound hypotonia, medically refractory seizures, severe developmental delay followed by neuroregression, and progressive cerebral and cerebellar atrophy on neuroimaging. Autonomic dysfunction is common and contributes to temperature instability, feeding difficulties, and recurrent hypoglycemia. Extra-neurological features arise primarily from connective-tissue deficits and copper-dependent enzymatic failure. Infants may display the characteristic brittle, sparse, lightly pigmented kinky hair, along with hypopigmented and lax skin, joint hypermobility, and congenital or acquired hernias. Vascular abnormalities, including arterial tortuosity and aneurysm formation, are frequent and contribute substantially to morbidity and mortality. Additional findings include bladder and gastrointestinal diverticula, persistent neonatal jaundice, recurrent infections, and failure to thrive. Without treatment, classic MD is fulminant, with most affected children dying by two to three years of age [20,23,24,25,26,27,28,29,30,31,32,33,34,35,36,37,38].

Biochemically, MD is characterized by low serum copper and Cp concentrations, which may superficially resemble biochemical findings in WD [38]. However, a key distinction is that urinary copper excretion is low, not elevated, and hepatic copper content is reduced, reflecting global systemic copper deficiency except within enterocytes, where copper accumulates due to impaired export. Diagnostic complexity arises in neonates younger than two months, as serum copper levels are physiologically low in this age group. In such cases, catecholamine metabolite analysis is particularly informative: elevated serum or cerebrospinal fluid dopamine-β-hydroxylase substrates and increased dopamine-to-norepinephrine or 3,4-dihydroxyphenylacetic acid (DOPAC)-to-3,4-dihydroxyphenylglycol (DHPG) ratios strongly support MD when routine biochemical markers are inconclusive. Definitive diagnosis relies on molecular genetic testing, with *ATP7A* sequencing or copy-number analysis providing confirmation [38,39].

For many years, management of MD consisted solely of supportive care aimed at seizure control, nutritional optimization, management of spasticity, hernias, and vascular complications [39,40,41,42,43]. Significant progress has been made with copper replacement therapy, particularly subcutaneous copper histidinate (CUTX-101), which bypasses the defective intestinal transport pathway and delivers bioavailable copper directly into systemic circulation. Early studies demonstrated that treatment initiated during the neonatal period improves survival, enhances neurodevelopmental outcomes, and increases copper concentrations in serum and cerebrospinal fluid. More recent data suggest that early administration of CUTX-101 may reduce mortality by nearly 80% compared with historical untreated cohorts, with early-treated infants achieving markedly prolonged survival. Although CUTX-101 has received orphan-drug designation and priority review from regulatory agencies, it remains under late-stage evaluation and is not yet universally approved as standard therapy [18,19].

#### 2.1.2. Occipital Horn Syndrome

Occipital horn syndrome (OHS; OMIM #304150), historically referred to as Ehlers–Danlos syndrome type IX, represents the mildest allelic variant within the *ATP7A*-related copper-transport disorders. OHS results from hypomorphic *ATP7A* (OMIM #300011) mutations—most commonly exon-skipping or missense variants—that partially preserve *ATP7A*-mediated copper transport [18,19,44,45,46]. This residual functional activity allows limited copper delivery to the central nervous system, accounting for the substantially attenuated neurological involvement compared with MD [45,46].

Clinically, OHS presents with a broad but generally non-life-threatening constellation of symptoms. The hallmark feature is the presence of wedge-shaped calcifications at the insertions of the trapezius and sternocleidomastoid muscles on the occipital bone. These “occipital horns” typically become apparent in late childhood and are readily identified on plain radiographs. Connective-tissue abnormalities are common and include skin and joint laxity, coarse and brittle hair, vascular tortuosity, bladder and intestinal diverticula, and a tendency toward hernia formation. Diverticula may contribute to obstructive or infectious uropathy, recurrent urinary tract infections, abdominal pain, and diarrhea. Vascular involvement—manifesting as arterial tortuosity, aneurysm formation, or, less commonly, hemorrhage from medium-sized arteries—adds to the clinical burden. Neurological manifestations are usually mild and include delayed neurodevelopment, dysautonomia, hypotonia, and impaired thermoregulation [18,19,45,46].

Biochemically, OHS is characterized by reduced serum copper and Cp concentrations, resembling the profiles observed in both MD and WD. However, a key distinguishing feature is that urinary copper excretion is typically normal, reflecting the distinct pathophysiological nature of the copper-handling defect. Catecholamine metabolite abnormalities in serum or cerebrospinal fluid—similar to those seen in MD but less pronounced—provide additional diagnostic evidence. Definitive diagnosis relies on molecular genetic testing of the *ATP7A* gene [18].

At present, no disease-modifying therapy exists for OHS. Management is supportive and requires multidisciplinary care addressing recurrent infections, urological complications related to diverticula, orthopedic issues, and vascular abnormalities. Selected individuals may derive benefit from copper supplementation, as the partially preserved *ATP7A* activity in OHS allows some degree of copper utilization, an effect not achievable in classic MD. Although the clinical impact is modest, early copper replacement may ameliorate autonomic dysfunction and support neurodevelopmental progress in some patients.

#### 2.1.3. X-Linked Distal Hereditary Motor Neuronopathy

X-linked distal hereditary motor neuronopathy (HMNX; OMIM #300489) represents the rarest and clinically mildest phenotype within the spectrum of *ATP7A*-related neurodegeneration [47,48,49,50]. In contrast to MD and OHS, HMNX exhibits a distinct clinical and biochemical profile. This phenotype results from specific missense variants in the *ATP7A* gene (OMIM #300011), typically involving amino acid substitutions within or adjacent to the carboxyl-terminal transmembrane domains. These variants preserve approximately 60–70% of normal *ATP7A* function, allowing maintenance of systemic copper homeostasis. Consequently, serum copper and Cp concentrations remain within normal limits, and systemic features typical of classical *ATP7A* deficiency are absent. The selective vulnerability of motor neurons suggests that these cells may be particularly sensitive to subtle disturbances in *ATP7A*-dependent intracellular copper trafficking [51,52].

Clinically, HMNX manifests as a slowly progressive, predominantly distal motor neuropathy. Patients develop weakness and atrophy of intrinsic hand and foot muscles, foot drop, pes cavus, and reduced or absent deep tendon reflexes. Sensory examination is typically normal, and pain is uncommon. Electrophysiological studies reveal an axonal motor neuropathy closely resembling Charcot–Marie–Tooth disease type 2. Age at onset is variable, ranging from early childhood to late adulthood, although most individuals present between 15 and 30 years of age. The disease progresses gradually, and substantial disability typically emerges only after many years [47,48,49,50,51,52].

Diagnostic evaluation is based on the characteristic clinical phenotype, nerve conduction studies demonstrating motor axonopathy, and confirmatory molecular testing of *ATP7A*. As biochemical copper indices are entirely normal in this disorder, reliance on serum or urinary copper studies may be misleading and highlights the necessity of genetic testing in individuals with unexplained distal motor neuropathy.

Currently, no disease-modifying therapy exists for HMNX. Management is supportive and includes physiotherapy, orthotic interventions, and symptomatic measures aimed at improving gait and functional performance [18].

### 2.2. MEDNIK Syndrome

MEDNIK syndrome (OMIM #609313)—an acronym for intellectual disability, enteropathy, deafness, peripheral neuropathy, ichthyosis, and keratoderma—is an exceptionally rare autosomal recessive disorder caused by pathogenic variants in the *AP1S1* gene (OMIM *603531), located on chromosome 7q22.1 [53,54,55]. *AP1S1* encodes a subunit of the adaptor protein complex AP-1, one of the five major adaptor complexes responsible for vesicular trafficking between the trans-Golgi network and endosomal compartments. AP-1 plays a critical role in intracellular sorting and transport of multiple transmembrane proteins, including the copper-transporting ATPases *ATP7A* and *ATP7B* [18,19,53].

As a result, *AP1S1* mutations impair both intestinal copper absorption and intracellular copper trafficking, generating a hybrid biochemical and clinical phenotype that shares key features of MD and WD [53,54,55]. Pathophysiology reflects a dual mechanism: defective copper delivery to cuproenzymes, mimicking MD, and impaired copper excretion with hepatic accumulation, paralleling WD. This combined disturbance produces a broad multisystemic phenotype. Neurological abnormalities include developmental delay, cognitive impairment, sensorineural hearing loss, and peripheral neuropathy. Skeletal features may involve delayed ossification and characteristic dysmorphism, such as a high forehead and facial features resembling Down syndrome. Cutaneous manifestations are prominent and include lamellar or erythrodermic ichthyosis, keratoderma, hypopigmentation of skin and hair, and brittle hair. Enteropathy with chronic diarrhea and malabsorption is common, substantially contributing to failure to thrive in early childhood [53,54,55]. Hepatic involvement in MEDNIK is notably similar to WD. Many patients present with hepatomegaly, elevated transaminases, and progression toward chronic liver injury. Brain magnetic resonance imaging (MRI) often reveals symmetric T2 hyperintensities in the basal ganglia—particularly the caudate nuclei and putamina—a pattern strongly reminiscent of WD and a potential diagnostic pitfall. Copper studies add further complexity: patients typically show reduced serum Cp and low total serum copper but, importantly, increased urinary copper excretion and elevated hepatic copper content—biochemical hallmarks that substantially overlap with WD. Without careful clinical assessment, these findings may lead to misdiagnosis and inappropriate initiation of anti-copper therapy [53,54,55]. Nevertheless, the distinctive constellation of MEDNIK features—particularly the dermatologic findings, dysmorphic traits, ichthyosis, hearing loss, developmental delay, and the full acronym pattern—should prompt consideration of this alternative copper metabolism disorder. Definitive diagnosis requires molecular confirmation of *AP1S1* variants.

To date, only 16 patients with MEDNIK syndrome have been described in the literature, underscoring its extreme rarity. The prognosis is generally poor, with approximately half of the reported patients deceased. No disease-modifying therapy exists, and management remains supportive, targeting dermatologic care, nutritional deficiencies, neurological complications, and hepatic involvement. Trials of zinc acetate—aimed at modulating intestinal copper absorption—have produced inconsistent results, and no consensus therapeutic approach has been established [18,19,53,54,55].

### 2.3. Primary Familial Intrahepatic Cholestasis Type 3

Progressive familial intrahepatic cholestasis type 3 (PFIC3; OMIM #601847) is a rare autosomal recessive disorder, estimated to occur in 1–2 per 100,000 live births, caused by pathogenic variants in the *ABCB4* gene (OMIM *171060) located on chromosome 7q21 [56,57,58,59,60,61,62,63,64,65,66]. *ABCB4* encodes multidrug resistance protein 3 (MDR3), a canalicular phospholipid flippase responsible for translocating phosphatidylcholine from the inner to the outer leaflet of the canalicular membrane. Phosphatidylcholine combines with bile salts to form mixed micelles, which protect the biliary epithelium from the detergent effects of bile acids. Loss or severe impairment of MDR3 function disrupts phosphatidylcholine secretion, leaving the biliary tree vulnerable to bile acid-mediated injury, ultimately resulting in chronic cholestasis, progressive hepatocellular damage, biliary fibrosis, and cirrhosis [56,57,58,59,60,61,62,63,64,65,66].

Clinically, PFIC3 typically presents in early childhood with cholestasis, pruritus, hepatomegaly, and elevated γ-glutamyl transferase (GGT), although adult-onset cases have also been described. The disease is progressive, frequently necessitating liver transplantation in advanced stages.

**Figure 2:**
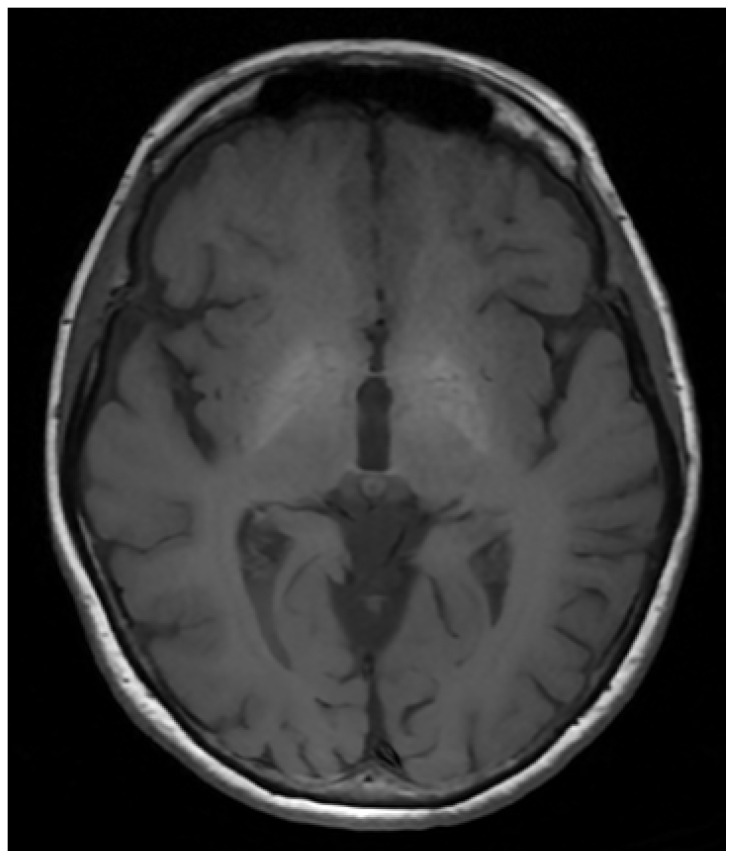
Brain MRI pathology in acquired hepatocerebral degeneration—symmetrical hyperintense signal in T1 sequence in globus pallidus (authors’ material from the Department of Neurology).

A notable diagnostic challenge arises from disturbances in copper metabolism. In chronic cholestasis, biliary copper excretion is impaired, leading to hepatic copper accumulation. Consequently, PFIC3 may present with markedly elevated hepatic copper content on biopsy—often exceeding the upper limit of normal—accompanied by reduced serum Cp and total serum copper and increased urinary copper excretion. This biochemical pattern closely resembles that observed in WD, potentially misleading clinicians, particularly when hepatic copper overload is pronounced. Several case reports describe patients initially misdiagnosed and treated as WD based on copper studies and liver biopsy findings [58,59,61,62,64,65,66]. Unlike WD, however, PFIC3 typically lacks neurological manifestations, and characteristic brain MRI findings of WD are absent. In rare cases, neurological symptoms may arise due to acquired hepatocerebral degeneration (AHD), a complication of chronic liver dysfunction. In AHD, bilateral T1 hyperintensities appear in the globus pallidus due to manganese deposition, superficially mimicking basal ganglia involvement in WD but reflecting a distinct pathophysiological mechanism (Figure 2) [67,68,69,70]. Misinterpretation of these MRI features, combined with elevated Leipzig scores driven by copper abnormalities, has contributed to erroneous WD diagnoses and inappropriate anti-copper therapy [58,59,61,62,64,65,66]. Definitive diagnosis of PFIC3 relies on molecular confirmation of *ABCB4* mutations.

Management includes ursodeoxycholic acid to improve cholestasis, while patients with advanced liver disease may require liver transplantation [56].

### 2.4. Huppke-Brendel Syndrome

Huppke–Brendel syndrome (HPBDS; OMIM #614416) is an autosomal recessive neurodevelopmental disorder caused by biallelic pathogenic variants in *SLC33A1* (OMIM *615849), located on chromosome 3q25.31. *SLC33A1* encodes acetyl-CoA transporter 1 (AT-1), an endoplasmic reticulum (ER) membrane protein that imports acetyl-CoA into the ER lumen [71,72]. AT-1 is essential for lysine acetylation of ER-resident proteins and for maintaining autophagy-dependent ER-associated degradation pathways that clear misfolded or aggregated proteins. Loss of AT-1 function leads to global impairment of ER protein acetylation, including that of Cp—a glycoprotein whose proper acetylation is required for stability and secretion. As a result, Cp secretion is markedly reduced, a phenomenon demonstrated experimentally in Human Hepatocellular Carcinoma G2 hepatocyte (HepG2) models [71,72]. The consequent low serum Cp and total serum copper may closely resemble the biochemical pattern of WD. Some patients additionally exhibit increased urinary copper excretion or abnormalities on radiocopper incorporation studies due to the profound deficiency of circulating Cp.

Importantly, despite these copper-related laboratory abnormalities, HPBDS does not produce copper toxicity. Liver copper concentrations remain normal, and patients do not develop hepatic copper overload, cirrhosis, or basal ganglia degeneration. Thus, the biochemical phenotype reflects defective Cp biosynthesis rather than impaired copper handling, distinguishing HPBDS from WD and other copper toxicoses. Clinically, HPBDS manifests as a severe early-onset neurodevelopmental disorder. Hallmark features include bilateral congenital cataracts, sensorineural hearing loss, severe global developmental delay, axial hypotonia, and markedly impaired psychomotor and cognitive development. Brain MRI typically demonstrates diffuse hypomyelination and generalized cerebral atrophy.

To date, eleven individuals have been reported: ten children, all of whom died between 6 months and 6 years of age, and one adult who presented with spastic ataxia and a unilateral parkinsonian tremor. Notably, this adult had been misdiagnosed with WD for 25 years and treated with zinc salts despite lacking any evidence of copper overload, illustrating the diagnostic pitfalls associated with the biochemical profile [72].

Diagnosis requires recognition of the characteristic neurodevelopmental phenotype, copper metabolism abnormalities, and neuroimaging findings, followed by molecular confirmation of *SLC33A1* mutations. Meticulous evaluation is essential to avoid erroneous classification as WD and the ensuing inappropriate anti-copper therapy [71,72].

There is currently no disease-modifying treatment for HPBDS. Management is supportive and directed toward the predominant sensory and neurological deficits. Interventions include cataract removal, hearing amplification, physiotherapy, and nutritional support. Experimental therapeutic attempts—such as ketogenic diet or N-acetylcysteine supplementation—have been described, but evidence for clinical benefit remains limited [71,72].

### 2.5. Aceruloplasminemia

Aceruloplasminemia (ACP; OMIM #604290) belongs to the heterogeneous group of rare inherited disorders collectively referred to as Neurodegeneration with Brain Iron Accumulation (NBIA), in which abnormal cerebral iron deposition is a defining feature [73,74,75]. It is a rare autosomal recessive disorder caused by pathogenic variants in the *CP* gene (OMIM *116860), located on chromosome 3q24–q25 [73,74,75,76,77]. The *CP* gene comprises 20 exons and encodes Cp, a multi-copper oxidase with dual functions: as a copper-transporting protein in serum and as a ferroxidase catalyzing the conversion of ferrous (Fe^2+^) to ferric (Fe^3+^) iron [78,79,80,81]. This oxidation step is essential for iron binding to transferrin, facilitating systemic circulation and cellular uptake via transferrin receptors [78,79,80,81]. Pathogenic *CP* variants result in markedly reduced or absent Cp activity and protein levels, causing impaired iron efflux, reduced circulating iron, defective erythropoiesis, intracellular iron accumulation, and subsequent tissue injury [78,79,80,81]. Organs predominantly affected include the brain, liver, pancreas, heart, and retina [78,79,80,81].

ACP was first described in 1987 by Miyajima et al., who reported a 52-year-old Japanese woman with progressive extrapyramidal symptoms, blepharospasm, retinal pigmentary degeneration, and diabetes mellitus [75]. The true prevalence of ACP is likely underestimated. The reported prevalence in Japan was approximately 1 per 2 million people based on a 1999 study with a small population sample [82]. A subsequent study estimating the lifetime risk of NBIA disorders calculated a prevalence of ~1 per 2.5 million globally [83].

Pathophysiology reflects tissue damage secondary to iron deposition, combined with paradoxical intracellular iron deficiency [84,85]. This mechanism underlies the clinical triad of ACP: diabetes mellitus, retinal pigmentary degeneration, and neurological manifestations, which include cerebellar, extrapyramidal, behavioral, and cognitive symptoms [73,74,75,76,77]. Laboratory findings form a biochemical triad: microcytic anemia, elevated serum ferritin, and low transferrin saturation [73,74,75,76,77,78]. Systemic features typically precede neurological symptoms by about a decade, presenting as mild microcytic anemia, low serum iron, hyperferritinemia, low transferrin saturation, diabetes mellitus, and retinal degeneration [82]. Neurological involvement encompasses cerebellar signs (dysarthria, truncal and limb ataxia) and involuntary movements (dystonia, chorea, tremor, blepharospasm) [74,78,81,83], while psychiatric manifestations may include cognitive impairment, apathy, and behavioral disturbances [74,78,81,83]. Additional systemic involvement can include hepatic iron accumulation and cardiac complications [78]. Animal studies suggest that early cellular dysfunction in ACP is driven by intracellular iron deficiency, whereas neurodegeneration emerges later secondary to iron overload and oxidative stress [16,84,85]. This mechanism explains the typical age-dependent course: microcytic anemia presents first (mean age 29.7 years), followed by diabetes mellitus (mean age 37.3 years), and neuropsychiatric symptoms (mean age 50.7 years) [73].

**Figure 3:**
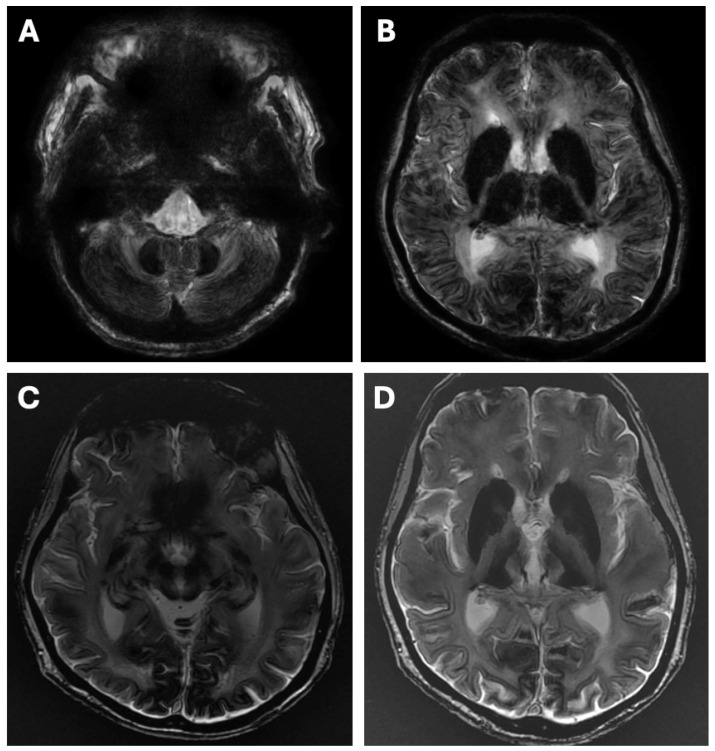
Brain MRI pathology in aceruloplasminemia—symmetrical hypointense signal in caudate, globus pallidus, putamen, thalamus, red nucleus, dentate in susceptibility-weighted imaging (SWI) sequences (**A**,**B**) and T2* sequences (**C**,**D**) (authors’ material from the Department of Neurology).

Diagnosis is challenging. The combination of the biochemical triad with low serum Cp is highly suggestive of ACP [73,74,75,76,77,78]. Brain MRI typically reveals symmetrical lesions involving the dentate nuclei, basal ganglia, and thalami, consistent with iron accumulation [86] (Figure 3).

Treatment options remain limited. Iron chelation therapy (deferasirox, deferoxamine, deferiprone) is considered first-line [87]. Combined strategies, such as iron chelation with fresh frozen plasma or phlebotomy, have shown partial benefit in selected cases, but overall efficacy remains uncertain [74,88,89]. Tetracyclines (e.g., minocycline) have been explored for their iron-chelating capacity and blood–brain barrier penetration [90]. Modest clinical improvement has also been reported with antioxidant therapy, including vitamin E and zinc sulfate [91].

### 2.6. Congenital Disorders of Glycosylation

Congenital disorders of glycosylation (CDG) constitute a large, heterogeneous group of over 160 rare metabolic diseases, with an estimated prevalence of approximately 1/10 000 [92,93,94,95,96,97,98]. Most CDG are inherited in an autosomal recessive manner, although autosomal dominant and X-linked forms have also been described. Nomenclature follows the affected gene name (non-italicized) followed by “-CDG” (e.g., CAD-CDG, carbamoyl-phosphate synthetase 2, aspartate transcarbamylase, and dihydroorotase deficiency) [92,93,94,95,96,97,98]. These disorders result from defects in the glycosylation process, leading to improper attachment of carbohydrate chains to proteins and lipids. Because glycosylation is critical for the function of multiple organ systems, CDG frequently present with multisystem phenotypes, including developmental delay, failure to thrive, hypotonia, ataxia, seizures, dysmorphic features, and hepatic involvement resembling WD [97,98].

Liver disease occurs in approximately 22% of CDG cases and exhibits a broad spectrum, ranging from simple steatosis to fibrosis or portal inflammation. Clinically, manifestations vary from mild elevations in liver enzymes to advanced decompensated liver failure. Coagulopathy may arise secondary to hepatic dysfunction or as a direct consequence of abnormal glycosylation of coagulation factors. In several CDG subtypes affecting both the brain and liver, disturbances in copper metabolism may mimic WD, including low serum copper and Cp, increased urinary copper excretion, and elevated hepatic copper content. These abnormalities are thought to result from defective glycosylation of Cp, which compromises its stability and secretion [92,93].

Serum carbohydrate-deficient transferrin (CDT) testing is typically employed as a first-line screening tool when CDG is suspected, although it primarily detects N-glycosylation defects associated with sialic acid deficiencies. Definitive diagnosis requires molecular genetic testing [94].

Management of CDG is largely supportive, with disease-specific therapies available only for a few subtypes, such as oral uridine supplementation in CAD-CDG or oral mannose in MPI-CDG (mannosephosphate isomerase deficiency) [98].

### 2.7. Acquired Copper Deficiency Syndromes

Copper is an essential trace element that functions as a cofactor for numerous cuproenzymes critical for neurological and systemic function [18,19]. These include superoxide dismutase (SOD1), dopamine-β-hydroxylase (dopamine synthesis), and peptidylglycine α-amidating monooxygenase (PAM). Copper also contributes to organismal growth and tissue maintenance, including collagen and elastin crosslinking (lysyl oxidase), melanin synthesis (tyrosinase), iron efflux (Cp, hephaestin, heme oxygenase) and numerous other essential processes. Consequently, copper deficiency disrupts enzymatic activity and may lead to severe neurological, hematological, and multisystem disorders that can mimic WD [18,19,99,100,101,102,103,104,105].

The primary causes of acquired copper deficiency (ACD) include bariatric surgery, with 15–19% of patients developing hypocupremia within the first 15 months postoperatively, depending on the surgical procedure, and malnutrition syndromes impairing copper absorption [104,105]. Additional causes encompass nephrotic syndrome or renal dialysis, which result in urinary copper loss, and excessive zinc intake, which competitively inhibits copper absorption. Less commonly, iron supplementation or disulfiram use may contribute [103,104,105]. Paradoxically, copper deficiency can also develop in approximately 1% of WD patients receiving long-term anti-copper therapy, typically with zinc salts, leading to neurological and hematological deterioration that may resemble the progression of WD [99,100,101,102].

Clinically, ACD syndromes are characterized by hematological abnormalities, including anemia, neutropenia, and leukopenia, alongside neurological deficits, often presenting as posterior cord syndrome with ataxia, spastic paraparesis, and axonal-demyelinating polyneuropathy. Additional manifestations may include brain demyelination, epileptic seizures, and optic neuropathy [99,100,101,102,103,104,105].

Diagnosis relies on careful assessment of clinical features, medical history, and copper metabolism parameters. Both ACD syndromes and WD may present with low total serum copper and Cp levels; however, in ACD syndromes, NCC is low, and daily urinary copper excretion is typically reduced, distinguishing it from WD [1,2,3,99,100,101,102].

Management involves copper supplementation and correction of underlying causes. In WD patients who develop ACD, temporary interruption of anti-copper therapy or switching from zinc salts to chelators may be necessary. Prognosis is generally favorable: hematological abnormalities usually resolve, and neurological symptoms often improve, although approximately one-third of patients may experience persistent neurological deficits despite treatment [101,102].

## 3. Emerging Concepts

Recent advances in metabolomics, proteomics, and molecular diagnostics have opened new avenues for differentiating WD from conditions that mimic WD [18,19]. Traditional markers, such as total serum copper and ceruloplasmin, often lack specificity, particularly in rare disorders like HPBDS or MEDNIK. Consequently, attention has shifted toward more subtle functional indicators. For example, catecholamine metabolite ratios (e.g., DOPAC/DHPG) in MD can reveal copper deficiency in the central nervous system before standard biochemical markers become informative. Similarly, in congenital disorders of glycosylation and MEDNIK syndrome, detailed glycoprotein profiling can identify abnormal Cp modifications, providing additional clues to distinguish these conditions from WD. Refinements in NCC measurement also enable the detection of subtle alterations in copper metabolism in phenocopies, improving diagnostic accuracy. Molecular testing now allows precise confirmation through sequencing of *ATP7A*, *SLC33A1*, *AP1S1*, and *CP* genes, which is crucial for timely and individualized therapeutic decisions.

Parallel to diagnostic progress, experimental therapies are emerging. CUTX-101 has demonstrated promising results in MD, improving survival and neurodevelopmental outcomes in neonates. In HPBDS, supportive interventions aimed at enhancing metabolic efficiency and reducing oxidative stress, including ketogenic diets or N-acetylcysteine supplementation, are under investigation. In aceruloplasminemia, combinatory approaches such as iron chelation with antioxidant therapy are being explored. Moreover, molecular strategies—including gene therapy, protein chaperones, and small molecules targeting enzymatic function—offer potential for transformative therapies in selected phenocopies, marking a new era of precision medicine in copper metabolism disorders.

## 4. Epidemiology and Diagnostic Challenges

Conditions that mimic WD are exceedingly rare but carry significant clinical implications due to the risk of misdiagnosis and inappropriate therapy. MD occurs in approximately 1 in 100,000 live births. MEDNIK syndrome has been reported in only 16 patients to date, HPBDS in 11, and *ATP7A*-related phenotypes such as OHS or HMNX are observed sporadically [18,19].

Misdiagnosis frequently leads to prolonged treatment with D-penicillamine or zinc salts in patients who do not benefit, sometimes spanning years or even decades, and in certain cases causing clinical deterioration. These experiences underscore the critical need for a thorough differential diagnosis, integrating clinical features, detailed biochemical assessment, and genetic testing.

Differentiation is complicated by partial overlap of phenotypes: neurological, hepatic, hematological, and psychiatric manifestations may closely resemble WD. As highlighted in Table 2 and Table 3 and Figure 1, Figure 2 and Figure 3, a comprehensive evaluation—including brain imaging, review of developmental and clinical history, and recognition of pathognomonic features such as occipital horns, lamellar ichthyosis, or dysmorphic traits—is essential to avoid diagnostic errors.

Although conditions that mimic WD are rare, their identification is clinically crucial: it prevents potentially harmful chelation therapy in non-WD patients and allows timely initiation of experimental or supportive interventions in conditions that may respond to early treatment, such as MD or HPBDS. Accurate recognition and characterization of these disorders thus hold direct translational relevance, enabling optimized patient care and more effective therapeutic decision-making.

## 5. Clinical Pearls

Accurate differentiation of WD from conditions that mimic WD is essential for ensuring appropriate management and avoiding unnecessary or potentially harmful therapy. Although classical biochemical markers—such as low serum Cp, reduced total serum copper, and elevated 24 h urinary copper excretion—remain central to WD diagnosis, phenocopies can present with overlapping laboratory profiles, making clinical context indispensable.

In practice, careful assessment of the patient’s history and clinical features often provides the first clues. The age of onset, developmental trajectory, and progression of symptoms may point toward a specific phenocopy rather than WD. For example, infants presenting with early neurodegeneration, hypotonia, or seizures are more suggestive of MD, while the presence of occipital horns in OHS, ichthyosis, and keratoderma in MEDNIK syndrome, or dysmorphic features in CDG further guide differential diagnosis.

Laboratory evaluation requires nuanced interpretation. In WD, NCC is typically elevated, whereas *ATP7A*- or *SLC33A1*-related disorders often show low or normal NCC. Urinary copper excretion, though high in WD and certain MEDNIK or CDG subtypes, may be low in MD or ACD syndromes. Additional biochemical markers—such as catecholamine metabolite ratios, CDT, or broader glycoprotein profiles—can help clarify ambiguous cases. Imaging and functional studies add another layer of diagnostic discrimination. Brain MRI may demonstrate basal ganglia involvement in WD, symmetric T2 hyperintensities in MEDNIK, or diffuse hypomyelination in HPBDS. Ophthalmologic examination remains valuable, particularly for detecting Kayser–Fleischer rings when neurological symptoms predominate. Molecular testing provides definitive confirmation and is indispensable when clinical and laboratory findings are inconsistent. Stepwise genetic approaches, beginning with targeted gene panels based on suspected phenocopy and escalating to whole-exome sequencing when needed, can identify pathogenic variants in *ATP7B* for WD or in *ATP7A*, *AP1S1*, *SLC33A1*, *CP*, and relevant glycosylation genes for conditions that mimic WD.

Therapeutically, avoiding empirical chelation in patients without confirmed WD is critical, as inappropriate treatment can induce copper deficiency and worsen neurological or hematological outcomes. Conversely, recognition of phenocopies with emerging disease-modifying therapies—such as CUTX-101 in MD—allows early intervention that may improve survival and neurodevelopmental outcomes.

Overall, integrating clinical, biochemical, imaging, and genetic information within a structured diagnostic framework—summarized in Table 2 and Table 3 and Figure 1, Figure 2 and Figure 3—facilitates accurate differentiation between WD and conditions that mimic WD. This approach minimizes the risk of misdiagnosis, guides appropriate management, and identifies candidates for novel or experimental therapies across both pediatric and adult populations.

## 6. Translational Perspective

The precise differentiation of WD from conditions that mimic WD carries profound translational implications, both for patient care and for the development of novel therapeutic strategies [1,2,3]. Misdiagnosis can lead not only to unnecessary or potentially harmful chelation therapy but also to missed opportunities for early intervention in disorders with emerging disease-modifying treatments. In this context, translational approaches emphasize the integration of molecular genetics, mechanistic understanding of copper and related metabolic pathways, and individualized patient care.

Accurate classification allows clinicians to tailor interventions according to the underlying pathophysiology. For example, early recognition of MD permits prompt administration of CUTX-101, which bypasses defective intestinal transport and directly supplies bioavailable copper to tissues, improving both survival and neurodevelopment. Similarly, the identification of HPBDS or ACP opens avenues for supportive strategies that target specific molecular deficits, such as experimental modulation of ER protein acetylation or iron chelation therapies. In congenital glycosylation disorders and MEDNIK syndrome, understanding defective protein processing or impaired copper trafficking informs future avenues for substrate supplementation or targeted metabolic interventions [18,19].

Beyond individual patient care, translational research in conditions that mimic WD offers broader insights into copper and iron homeostasis, glycosylation biology, and neurodevelopmental vulnerability. Investigations into novel biomarkers—whether metabolomic, glycomic, or copper-binding protein profiles—have the potential to improve early detection, refine risk stratification, and distinguish subtle phenocopies before irreversible organ damage occurs. Advances in gene therapy, chaperone molecules, and small-molecule modulators further highlight the promise of precision medicine in these rare disorders, emphasizing that accurate diagnosis is a prerequisite for therapeutic innovation [18,19].

In practice, translational application requires a multidisciplinary approach that combines careful clinical observation with state-of-the-art laboratory and imaging techniques, followed by targeted molecular testing. This framework ensures that patients receive the most appropriate therapy, avoids inappropriate chelation, and identifies candidates for clinical trials or experimental interventions. By bridging mechanistic understanding with clinical application, the translational perspective underscores the importance of precision diagnosis in optimizing outcomes and advancing care for patients with WD and conditions that mimic WD [1,2,3].

## 7. Conclusions

WD remains a prototypical inherited disorder of copper metabolism, yet a growing spectrum of conditions that mimic WD—both genetic and acquired—presents significant diagnostic challenges [1,2,3]. Accurate differentiation is critical, as misclassification can result in inappropriate anti-copper therapy, delayed treatment of the true underlying disorder, and potentially irreversible organ damage [10,12]. Conditions that mimic WD often exhibit overlapping clinical and biochemical features, including low serum ceruloplasmin, altered total serum copper, and hepatic copper accumulation, but careful assessment of subtle distinctions—such as age of onset, specific neurological signs, dermatologic features, or catecholamine and glycoprotein profiles—facilitates correct diagnosis (Table 2, Figure 1, Figure 2 and Figure 3).

Emerging concepts highlight the value of novel biomarkers and metabolomic approaches for distinguishing WD from its mimics [1]. Epidemiologically, most WD phenocopies are rare [18,19], yet misdiagnosis is not uncommon. From a practical perspective, structured diagnostic approaches—combining careful clinical assessment, interpretation of nuanced laboratory findings, imaging characterization, and targeted molecular testing—enhance diagnostic accuracy.

In translational terms, the accurate distinction between WD and its phenocopies maximizes patient safety, prevents unnecessary chelation, and optimizes timing for potentially disease-modifying interventions. This approach bridges the mechanistic understanding of copper metabolism with clinical application, allowing identification of patients who may benefit from emerging therapies, participate in clinical trials, or receive tailored supportive care.

In summary, the differential diagnosis of WD is no longer limited to classical clinical and biochemical parameters [1,3,18,19]. Incorporating emerging biomarkers, advanced imaging, and molecular diagnostics, alongside awareness of rare phenocopies, is essential for precision medicine. Integrating these insights into clinical practice promotes optimal patient outcomes, reduces mismanagement, and paves the way for therapeutic innovation in rare copper-related disorders.

## Figures and Tables

**Table 1:** Leipzig scoring system for the diagnosis of Wilson’s disease (developed 8th International Meeting on Wilson’s disease and Menkes disease, Leipzig 2001) [11].

Parameter	Criteria	Points
Kayser–Fleischer ring	Present	2
	Absent	0
Neuropsychiatric symptoms suggestive of WD (or typical brain MRI findings)	Present	2
	Absent	0
Coombs-negative hemolytic anemia	Present	1
	Absent	0
Serum ceruloplasmin (Cp) (nephelometric assay)	<10 mg/dL	2
	10–20 mg/dL	1
	>20 mg/dL (normal)	0
Daily urinary copper excretion	>2 × ULN or normal but >5 × ULN after D-penicillamine challenge (2 × 0.5 g)	2
	1–2 × ULN	1
	<50 µg/24 h (normal)	0
Hepatic copper concentration (liver biopsy)	>250 µg/g dry weight	2
	50–250 µg/g dry weight	1
	<50 µg/g dry weight	−1
Rhodanine-positive hepatocytes (if quantitative copper not available)	Present	1
	Absent	0
Mutation analysis in *ATP7B*	Pathogenic mutations on both alleles	4
	Pathogenic mutation on one allele	1
	No mutation detected	0

Interpretation of the score: ≥4 points—diagnosis of Wilson’s disease highly likely; 2–3 points—diagnosis probable (further investigations required); ≤1 point—diagnosis unlikely. Abbreviations: Cp—ceruloplasmin; MRI—magnetic resonance imaging; ULN—upper limit f normal; WD—Wilson’s disease.

**Table 2:** Disorders of copper metabolism mimicking Wilson’s disease—differential diagnosis.

	MD	Mednik Syndrome	PFIC3	HPBDS	ACP	CDG	ACD Syndromes
Presence of symptoms							
Neurological symptoms	+	+	−	−	+	+	+
Hepatopathy symptoms	−	−	+	−	+	+	depending on etiology
K-F ring	−	−	−	−	−	−	−
Brain MRI abnormalities in basal ganglia	+ rare, asymmetric hyperintensive signal in MRI T2 sequences involved the CN head and anterior PT	+ symmetric hyperintensive in MRI T2 sequences signal in the basal ganglia—particularly the NC and PT	+ rare reflecting manganese GP accumulation only as hyperintensive signal in GP in MRI T1 sequences	+ rare reflecting manganese GP accumulation only as hyperintensive signal in GP in MRI T1 sequences	+ symmetrical hypointense signal in NC, PT, GP, TH, DN, RN in MRI T2 sequences, rare with additionally mixed signal	−	−
Laboratory abnormalities							
ALT and AST serum level	normal	increased	increased	normal	increased	increased	increased
Total serum copper level	decreased	decreased	decreased	decreased	decreased	decreased	decreased
Serum Cp level	decreased	decreased	decreased	decreased	decreased/absent	decreased	decreased
Daily urinary copper excretion	low/normal	increased	increased	normal	normal	increased	low/normal
Liver copper content (biopsy)	decreased	increased	increased	increased	increased	increased	depending on etiology (increased/normal/decreased)

Abbreviations: ‘+’—present; ‘−’—absent; ACD—acquired copper deficiency syndrome; ACP—aceruloplasminemia; ALT—alanine aminotransferase; AST—aspartate aminotransferase; CN—caudate nucleus; CDG—congenital defects of glycosylation; Cp—ceruloplasmin; DN—dentate nucleus; GP—globus pallidus; HPBDS—Huppke–Brendel syndrome; MD—Menkes disease; MRI—magnetic resonance imaging; PFIC3—primary familial intrahepatic cholestasis type 3; PT—putamen; RN—red nucleus; TH—thalamus.

**Table 3:** Practical recommendations according to copper metabolism disorders.

Key Point	Practical Recommendation
Suspected WD with atypical presentation	Assess full clinical phenotype (hepatic, neurological, and systemic features) before initiating therapy.
Serum copper and Cp	Always interpret in context; low values may reflect copper deficiency syndromes rather than WD.
Urinary copper & NCC	Distinguish between true copper overload (WD) vs. reduced copper excretion (*ATP7A*-related disorders, ACD).
Neuroimaging	Evaluate basal ganglia, cerebellum, and white matter; consider iron deposition in ACP and cholestasis patterns in PFIC3.
Genetic testing	Sequence *ATP7B*, *ATP7A*, *SLC33A1*, *CP*, *ABCB4*, *AP1S1*, and relevant glycosylation genes in atypical cases.
Biochemical ratios	Consider DOPAC/DHPG, CDT, and Cp glycoforms as adjuncts for early differentiation.
Therapy initiation	Avoid empiric anti-copper therapy until WD is confirmed to prevent toxicity and delay inappropriate management.

Abbreviations: ACD—acquired copper deficiency syndrome; ACP—aceruloplasminemia; CDT—carbohydrate-deficient transferrin; Cp—ceruloplasmin; DOPAC/DHPG—dihydroxyphenylacetic acid-to-dihydroxyphenylglycol ratio; NCC—non-ceruloplasmin-bound copper; PFIC3—primary familial intrahepatic cholestasis type 3; WD—Wilson’s disease.

## Data Availability

No new data were created or analyzed in this study. Data sharing is not applicable to this article.
